# Detection of Prostate Cancer Antigen 3 and Prostate Cancer Susceptibility Candidate in Non-DRE Urine Improves Diagnosis of Prostate Cancer in Chinese Population

**DOI:** 10.1155/2020/3964615

**Published:** 2020-01-31

**Authors:** Lie-Fu Ye, Sha He, Xiaopei Wu, Shengying Jiang, Ruo-Chen Zhang, Ze-Song Yang, Fa-Wen Chen, Dan-Ling Pan, Dong Li, Gang Li

**Affiliations:** ^1^Department of Urology, Fujian Provincial Hospital, Fuzhou, Fujian, China; ^2^Shengli Clinical Medical College of Fujian Medical University, Fuzhou, Fujian, China; ^3^Fuzhou L-BioMedx Technology Co., Ltd., Fuzhou, Fujian, China; ^4^Clinical Laboratory, Fujian Provincial Hospital, Fuzhou, Fujian, China; ^5^Department of Pathology, Fujian Provincial Hospital, Fuzhou, Fujian, China; ^6^School of Medicine, Nankai University, Tianjin, China

## Abstract

Although prostate biopsy is the gold standard for the diagnosis of prostate cancer, it also leads to high incidence of negative biopsies and the diagnosis of clinically low-risk prostate cancer and the subsequent overtreatment. It remains an unmet need to discover new biomarkers in order to defer the unnecessary biopsies in clinical practice. In this study, we described a new method, LBXexo score, to measure the urine exosomal PCA3/PRAC expression from non-DRE urine as a noninvasive diagnosis to improve the detection rate in Chinese population with a low serum PSA level. First-voided urine samples were collected to isolate exosomes, and exosomal RNAs of PCA3 and PRAC were measured by quantitative reverse transcription PCR. A significant increase in exoPCA3/PRAC was observed in both any-grade and high-grade prostate cancer groups when compared with the biopsy-negative group. Receiver-operating characteristic curve analyses showed that the LBXexo score significantly improved diagnostic performance in predicting biopsy results, with AUCs of 0.723 (*p*=0.017) and 0.736 (*p*=0.038) for any-grade and high-grade (GS ≥ 7) prostate cancer, respectively. For high-grade cancer, LBXexo had the negative and positive predictive values of 100% and 27.59%, respectively, and could potentially avoid unnecessary biopsy. This is the first report in Chinese population that demonstrates the predictive value of the exosomal expression of PCA3 and PRAC derived from non-DRE urine in predicting prostate biopsy outcomes. It could be used in clinical practice to make a better informed biopsy decision and avoid unnecessary biopsies in Chinese population.

## 1. Introduction

Prostate cancer (PCa) is the most frequently diagnosed cancer and the second-leading cause of cancer death in male worldwide, with almost 1.3 million new cases diagnosed and 359,000 deaths in 2018 [1]. With economic growth and lifestyle change, prostate cancer is the most rapidly increasing male cancer in China, and it is currently the sixth common cancer in men with approximate 60,300 new cases in 2015 [2]. Prostate needle biopsy is still the gold standard for definitive diagnosis for patients with elevated serum PSA and suspicious digital rectal exam (DRE) together with age and family history. Although the PSA test significantly increases the PCa detection rate in those patients with considerably elevated serum PSA levels, the detection rate for low PSA levels, especially in patients with serum PSA levels between 4 and 10 ng/ml (grey zone), is generally low [3]. Due to low specificity of PSA and high prevalence of low-risk PCa, more than 70% of patients are unnecessarily biopsied [4]. Overdiagnosis also causes overtreatment of those low-risk PCa patients, and patients with indolent prostate cancer usually do not require treatment. As an invasive procedure, prostate needle biopsy is usually associated with pain and some severe complications such as infection, bleeding, and erectile dysfunction, and even mortality in some cases [5]. Therefore, there is a huge unmet need to reduce overdiagnosis of PCa caused by current clinical practices.

In recent years, great efforts have been taken to identify new biomarkers for PCa detection. Several PSA derivative tests such as % free PSA [6, 7], PSA velocity [8], and PSA precursor (2proPSA) have been used to improve the performance of the PSA test [9, 10]. Prostate cancer antigen 3 (PCA3), a long noncoding RNA that is highly expressed in prostate cancer compared with noncancerous prostate tissue, has been found to be independent of the prostate size and serum PSA level [11]. The PCA3 test, which measures PCA3 mRNA in urine samples after DRE and was approved by the FDA in 2012 as a risk assessment tool for prostate cancer, has been demonstrated as a useful tool to aid in guiding biopsy decision among men with prior negative prostate biopsies and improve the early PCa detection rate [12–14]. However, the ability of PCA3 to predict tumor aggressiveness remains controversial [15, 16]. The *TMRPSS2*-*ERG* gene fusion, a highly specific marker for PCa, has been reported in approximately 50% of Caucasian PCa patients [17, 18]. The combination of PCA3 and TMRPSS2-ERG measurement from post-DRE urine samples improves the sensitivity of PCa detection, reduces the number of biopsies, and adds significant predictive value to predict biopsy outcomes [19, 20].

Extracellular vesicles (EVs), lipid-enclosed particles released from live cells with the enriched macromolecules to reflect the cell of origin, have been recently reported as a useful liquid biopsy tool for various diseases [21–23]. Recent studies highlight the clinical application of urine-derived exosomes as a useful biomarker to discriminate indolent from clinically significant PCa [24, 25]. The ExoDx Prostate (IntelliScore) test, which analyzes the exosomal RNA expression of PCA3 and *TMRPSS2-ERG* from non-DRE urine, has been reported in two trials to have better sensitivity than the existing risk calculator to predict high-grade PCa (GS ≥ 7) at initial biopsy, and the IntelliScore test could defer unnecessary biopsies [26, 27].

Due to the heterogeneity of prostate cancer, the prevalence of the *TMPRSS2*-*ERG* fusion transcript varies by race. In contrast to high prevalence in Caucasians, the prevalence of *TMPRSS2*-*ERG* in Chinese population is very low, while the prevalence of PCA3 is high in Chinese population and the increased expression has been reported in Chinese prostate cancer patients [28]. Downregulation of the prostate cancer susceptibility candidate (PRAC) expression was found in cancerous tissue compared with BPH tissue, suggesting the correlation between the PRAC expression and prostate cancer development [29]. In this study, we reported for the first time the combination of PCA3 and PRAC gene expressions in exosomes isolated from non-DRE urine samples as a noninvasive test in predicting biopsy outcomes in Chinese population.

## 2. Materials and Methods

### 2.1. Study Population

From June 5, 2017, to May 29, 2018, 89 men scheduled for an initial or repeat prostate needle biopsy due to the elevated PSA level and/or a suspicious digital rectal examination (DRE) were invited to participate in this study at the Urology Unit of Fujian Provincial Hospital. All subjects provided written informed consent, and the protocol was approved by the Fujian Provincial Hospital Ethics Committee (K2017-09-065).

### 2.2. Sample Collection and Handling

First-catch prebiopsy urine samples (blinded to lab personnel) were collected and assigned an ID according to standard protocols. Samples were collected in a standard urine collection cup and delivered on ice to a central laboratory at Fujian Provincial Hospital for initial processing. The urine samples were centrifuged at 5000 rpm for 15 min at 4°C to remove debris, filtered through a 0.8 *μ*m syringe filter, and then stored in 30 ml aliquots at −80 C until further processing.

### 2.3. Extracellular Vesicle (EV) Isolation, RNA Extraction, and RT-PCR

A 25 ml aliquot of each sample was thawed in a water bath at 37°C for ∼15 minutes, followed by filtration through a 0.8 *μ*m syringe filter. The exosomes were isolated from filtrates by serial ultracentrifugation [30]. In brief, the urine samples were ultracentrifuged at 170,000 rpm for 2 hours and the pellets were then washed with ice-cold PBS for another 90 min at 4°C. The EV pellets were resuspended in 200 *μ*l of the PBS for further RNA extraction. Exosomal RNA was extracted from the EVs using the RNeasy Mini Kit (Qiagen, Germany).

### 2.4. RNA Reverse Transcription and qRT-PCR

The RNA (20 *μ*l) was reverse transcribed to cDNA using the RNA to cDNA EcoDry™ Premix kit (Clontech, USA) according to the manufacturer's protocol. qPCR was performed using the ABI ViiA 7 Real-Time PCR System: 95°C for 10 min, followed by 40 cycles of 95°C for 15 s and 60°C for 15 s. For each PCR, the following was added to the qPCR reaction mix: 4 *μ*l of preamplified cDNA, 12.5 *μ*l of Taqman™ Universal PCR Master Mix, 2.5 *μ*l of the primer, and 6 *μ*l of H_2_O, to make a total volume of 25 *μ*l. For the exosomal RNA gene relative expression, genes of interest were normalized to exoRNA KLK3 (C_t_ target gene: C_t_ KLK3).

### 2.5. Statistical Analysis

The Mann–Whitney *U* test was used to compare the expression of PCA3 and PCA3/PRAC between different groups. The receiver-operating characteristic curve analyses and statistical analysis were carried out using R statistical computing software version 3.5.1 (http://www.r-project.org).

## 3. Results

### 3.1. Patients' Characteristics

Non-DRE urine samples were collected from 89 subjects who were enrolled between June 5, 2017, and May 29, 2018, and did not take medications or hormones that are known to affect serum PSA levels at least 6 months prior to enrollment. Among these subjects, 57 met the eligibility criteria as “intended population” for this study with prebiopsy serum PSA < 20 ng/ml. First-voided urine samples were collected before biopsy for each subject. The median age was 65 years, and the median serum PSA was 9.59 ng/ml. The median number of prostate biopsy cores was 10. Most of the subjects had nonsuspicious DRE (70%) and had no prior biopsy (91.2%). Of all, 14 (24.6%) had prostate cancer and 43 (75.4%) were biopsy negative for prostate cancer (benign prostatic hyperplasia and prostatic intraepithelial neoplasia). Among the subjects with prostate cancer, 8 (57.1%) had Gleason score ≥7 and 6 (42.9%) had GS = 6. The clinical characteristics of subjects are summarized in Table 1.

### 3.2. The Urinary Exosomal Expression of PCA3, PRAC, and PCA3/PRAC in Prostate Cancer

**Figure 1:**
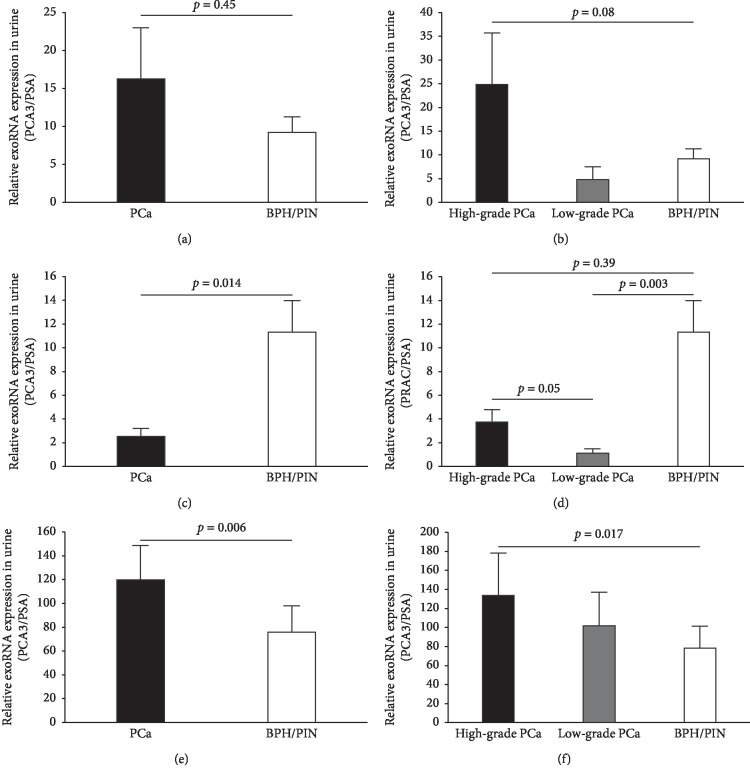
The exosomal RNA expression of PCA3/PRAC in non-DRE urine is increased in prostate cancer. (a) Urine RNA expression of PCA3 is normalized to PSA and is relatively high in the biopsy-positive (PCa) group, when compared with the biopsy-negative (BPH/PIN) group. (b) A higher expression of PCA3 is observed in the high-grade prostate cancer cohort. (c, d) Urine RNA expression of PRAC is normalized to PSA and is decreased in the biopsy-positive (PCa) group and in the low-grade prostate cancer group. (e, f) The exosomal PCA3/PRAC is significantly higher in the biopsy-positive (PCa) group and is increased by the Gleason score. High-grade PCa, Gleason score ≥7 (*n* = 8); low-grade PCa, Gleason score = 6 (*n* = 6); BPH, benign prostatic hyperplasia; PIN, prostatic intraepithelial neoplasia. The Mann–Whitney *U* test is used for statistical analysis. Data are presented as mean ± SEM.

Here, we aimed to measure the expression of PCA3, PRAC, and PSA in exosomes isolated from non-DRE urine samples by qRT-PCR. Exosomal mRNA transcripts of PCA3, PRAC, and PSA were detected in all 57 first-voided non-DRE urine samples. However, the exosomal expression of these genes was very low in plasma samples from these patients (data not shown). As shown in Figure 1, the urine exosomal PCA3 expression in prostate cancer patients was higher than that in biopsy-negative subjects but did not reach statistical significance (Figure 1(a); *p*=0.45). Increased exosomal PCA3 expression was more pronounced in high-grade prostate cancer (Figure 1(b); *p*=0.08). Similar to the decreased PRAC expression observed in prostate cancer tissue [29], the exosomal PRAC expression was decreased in the prostate cancer group, particularly in the low-grade prostate cancer group (Figure 1(d); *p*=0.003). Interestingly, the exosomal PRAC expression was significantly lower in the low-grade prostate cancer group compared with the high-grade prostate cancer group. Importantly, when the urine exosome PCA3/PRAC ratio was computed, we found that the PCA3/PRAC value in the prostate cancer group was significantly higher than that in the biopsy-negative group (Figure 1(e); *p*=0.006). In particular, the exosomal PCA3/PRAC value was significantly higher in the high-grade prostate cancer group (GS ≥ 7) than that in the biopsy-negative group (*p*=0.017), while PCA3/PRAC was comparable between low-grade and biopsy-negative groups (Figure 1(f)).

### 3.3. The Performance of Exosomal PCA3 and PCA3/PRAC in Predicting Biopsy Results

To assess the value of the exosomal PCA3/PRAC expression in predicting prostate cancer biopsy outcomes, we computed an LBXexo score for each subject. The LBXexo score is defined as a log-transformed PCA3 expression level relative to the PRAC expression. The receiver-operating characteristic (ROC) curve was used to determine the optimal cutoff point with a maximized Youden index for the LBXexo score. For both any-grade and high-grade prostate cancer, the LBXexo score (cutoff value of 5) showed good clinical performance to predict the biopsy outcome. Among 14 biopsy-positive subjects, an LBXexo cutoff point of 5 correctly identified 100% of high-grade prostate cancer subjects (GS ≥ 7) and missed two low-grade prostate cancer subjects (GS = 6) (Table 2). The LBXexo score demonstrated relatively greater sensitivity than PCA3 alone, with 85.7% vs. 71.4% for any-grade cancer and 100% vs. 87.5% for high-grade cancer. Importantly, the LBXexo score had a higher specificity than PCA3 in predicting biopsy results, with 60.5% vs. 34.9% and 57.1% vs. 36.7% for any-grade and high-grade prostate cancer, respectively (Table 3). In predicting any-grade cancer, the LBXexo score demonstrated greater PPV (41.38% vs. 26.32%) and NPV (92.86% vs. 78.95%) than PCA3 alone. In addition, among the 26 biopsy-negative subjects with the LBXexo score less than the cutoff point value, 12 subjects were followed up to collect one-year biopsy results and all 12 were biopsy negative for prostate cancer. These data demonstrated that the LBXexo score outperformed PCA3 alone in predicting the high-grade biopsy-positive outcome in Chinese population with PSA levels lower than 20 ng/ml, and the LBXexo score test could potentially reduce unnecessary repeat biopsies.

**Figure 2:**
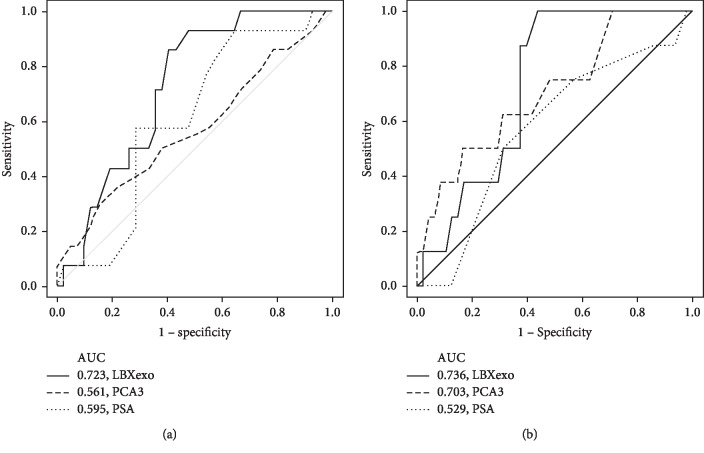
Urine exosomal PCA3/PRAC predicts the biopsy outcome. For each cohort, ROC curve analysis is performed for (a) any-grade and (b) high-grade prostate cancer to determine the AUC for LBXexo, PCA3 alone, and PSA test, respectively. ROC, receiver-operating characteristic; AUC, area under the curve.

We then further assessed the predictive performance of the LBXexo score and PCA3 alone to discriminate between biopsy-positive and benign prostate samples by ROC curve analyses and to compare with PSA. In line with low specificity in predicting biopsy-positive results, PCA3 alone had an AUC of 0.561 (95% CI 0.38–0.746, *p*=0.479). The AUC for the LBXexo score was 0.723 (95% CI 0.58–0.849, *p*=0.017) in predicting any-grade cancer, significantly higher than that of PCA3 alone and PSA (0.595, 95% CI 0.436–0.754, *p*=0.289). For high-grade prostate cancer, PCA3 alone and LBXexo score had an AUC value of 0.703 (95% CI 0.511–0.899, *p*=0.065) and 0.736 (95% CI 0.592–0.868, *p*=0.038), respectively, compared with the AUC of 0.529 (95% CI 0.334–0.723, *p*=0.797) for PSA (Figure 2; Table 4). These data demonstrated the predictive value of the LBXexo score to discriminate high-grade and any-grade prostate cancer.

The predictive accuracy of the LBXexo cutoff point of 5 was further evaluated in a validation cohort (Supplementary [Supplementary-material supplementary-material-1]). Among the 28 subjects that were newly enrolled with PSA levels lower than 20 ng/ml, the LBXexo score correctly identified 100% of high-grade prostate cancer subjects GS ≥ 7 and all low-grade prostate cancer subjects (GS = 6) (Table 5).

## 4. Discussion

This is the first study to report the diagnostic value of using non-DRE urine in Chinese population in predicting prostate biopsy outcomes. We identified a new urine index, LBXexo score, to measure the exosomal PCA3/PRAC expression from non-DRE urine samples and demonstrated its predictive value that is independent of serum PSA and could potentially reduce unnecessary biopsies in Chinese population with serum PSA levels below 20 ng/ml.

The gold standard for prostate cancer diagnosis is based on histopathological evaluation of prostate biopsy, an invasive procedure associated with discomfort, distress, and severe complications, due to the elevated serum PSA level and suspicious DRE. However, low specificity of PSA results in a high negative biopsy rate of 70% to 80%, especially in the patients with a low serum PSA level [31–33]. In addition, Macefield et al. reported that 75% of men with elevated serum PSA have benign biopsy findings [34]. Therefore, there is an unmet need to discover more sensitive biomarkers that can distinguish indolent from clinically significant prostate cancer and can reduce the number of repeated biopsies.

According to the European Association of Urology new guideline in 2017, additional diagnostic options are recommended for asymptomatic men with a normal DRE and a PSA between 2.0 and 10 ng/ml to avoid unnecessary biopsy, including additional serum- or urine-based tests, such as prostate health index blood (PHI), the 4-kallikrein blood test (4K score), and PCA3 and SelectMDx in urine samples after DRE. It has been reported that the PSA level in Asians is lower than that in Caucasians, and the detection rate for the same PSA range is much lower in Asian population [35]. Compared with the positive detection rate of 25% and 38% for the PSA range of 4–10 ng/ml and 10–20 ng/ml, respectively, in Caucasians [36], the prostate cancer detection rate in Chinese population is 16% and 25%, respectively [37]. In China, the majority of patients who underwent biopsy have PSA levels under 20 ng/ml [3]. Thus, it has been proposed that the expanded PSA range such as 4–20 ng/ml should be used as the grey zone for Chinese population [38]. In this study, we demonstrated the diagnostic value of the LBXexo score in predicting biopsy outcomes in Chinese population with serum PSA < 20 ng/ml.

The urine PCA3 test approved by the FDA in 2012 improves diagnostic accuracy to predict the repeated biopsy outcome [39]. However, the PCA3 test requires the DRE procedure, which is very subjective and requires office visits to perform prostate massage by a physician to collect enough cells for RNA analysis. The ExoDx Prostate (IntelliScore) test, measuring the exosomal gene expression of *TMPRSS2*-*ERG* and PCA3 in non-DRE urine samples, has recently demonstrated the independent diagnostic value to discriminate high-grade from low-grade and benign prostate disease in Caucasians, improve clinical identification of high-grade prostate cancer, and reduce the number of unnecessary biopsies [24, 26, 27].

Our data demonstrated that urine exosomal PCA3/PRAC was highly upregulated in a biopsy-positive cohort and accurately predicted all high-grade prostate cancer. In our intended study population with PSA < 20 ng/ml, the LBXexo score significantly improved diagnostic performance for both any-grade (AUC 0.723) and high-grade (AUC 0.736) biopsy subjects. The AUC values are comparable to the recently published data by using the ExoDx Prostate test in Caucasians, which had AUCs of 0.715 and 0.764 for any-grade and high-grade cancer, respectively [24]. The LBXexo score outperformed PCA3 alone in predicting any-grade prostate cancer, with a greater AUC by ROC curve analysis (0.723 vs. 0.561) and higher specificity (60.5% vs. 34.9%). Moreover, the LBXexo score demonstrated relatively greater diagnostic performance (AUC: 0.736 vs. 0.703) and better specificity (57.1% vs. 36.7%) than PCA3 alone for high-grade prostate cancer. Our findings also implicated that PCA3 alone was not effective and needs to be combined with the additional prostate-related gene to improve the detection performance of prostate cancer. Because the prevalence of the *TMPRSS2*-*ERG* fusion transcript in Chinese population is low, we detected the PRAC mRNA in urine-derived exosomes and discovered that the combination of urine exosome PCA3 and PRAC expressions enhanced the diagnostic value to predict the biopsy outcome for both any-grade and high-grade prostate cancer, which could potentially avoid unnecessary biopsy. These data reaffirm the diagnostic value of the LBXexo score in early detection which does not require a DRE procedure.

Although fine-needle biopsy is the standard procedure for prostate diagnosis and additional numbers of biopsy cores improve the detection rate [40, 41], its ability to reflect the pathology of the entire prostate is limited as prostate cancer is a multifocal disease containing the heterogeneous population of tumor cells. EVs are the nanoparticles secreted from all types of live cells into the circulation and are enriched with proteins and nucleic acids from donor cells, reflecting the snapshot of the physiopathology state of living organisms [42, 43]. Therefore, cancer-derived EVs isolated from biological fluids represent a valuable and noninvasive resource as liquid biopsy markers, which can improve diagnosis and prognosis [44, 45]. Several candidates have been identified in the prostate cancer-derived exosomes [46]. The mRNA of androgen receptor splice variant 7 (AR-V7) was detected in plasma-derived exosomes from patients with castration-resistant prostate cancer as a predictive biomarker of resistance to hormonal therapy [47]. The serum-derived exosomal expression of P-glycoprotein (P-gp), a drug efflux pump contributing to resistance to chemotherapy, was higher in docetaxel-resistant patients than in therapy-naive patients [48]. Exosomes are currently under extensive research as a novel drug delivery tool for cancer treatment [49]. Similar to the ExoDx Prostate (IntelliScore) test in Caucasians, we showed the clinical usefulness of exosomes isolated from non-DRE urine in predicting any-grade and high-grade prostate cancer in Chinese population with PSA levels lower than 20 ng/ml. Although all high-grade prostate cancer subjects were identified, the PCA3/PRAC test also identified 17 “false-positive” subjects in the biopsy-negative group (39.5%). It could be related to the heterogeneity of prostate cancer that biopsy core numbers used in this study could miss the clinically important prostate cancer, especially for those “false-positive” subjects with a high LBXexo score.

Despite the promising results, the current study has few limitations. First, the patient number is relatively small and the results need to be further validated in a larger cohort. A longer follow-up period should be considered in the future large cohort study to monitor biopsy-negative participants. Second, given the incomplete data of clinical staging and family history of these patients, we cannot include them as parts of the standard of care variables to compare the performance with the urine exosome test. Finally, it should be included in the future studies to compare the diagnostic performance of our urine exosome test with that of other assays currently available including prostate health index blood (PHI), the 4-kallikrein blood test (4K score), and the PCA3 test in urine samples after DRE.

## 5. Conclusions

This study demonstrates the LBXexo score, a novel noninvasive method utilizing first-voided, non-DRE urine, as a diagnostic tool that could independently predict any-grade and high-grade biopsy outcomes in Chinese population with serum PSA levels below 20 ng/ml. Once validated in a larger cohort, it could be used along with current prognostic indexes to make a better informed biopsy decision and avoid unnecessary biopsies in Chinese population. As a routine liquid biopsy option, a series of non-DRE urine exosome tests could provide benefits to track real-time progression of prostate cancer, which is not feasible by biopsy because of its invasiveness.

## Figures and Tables

**Table 1:** Subjects' clinical characteristics.

Characteristics	Median (range) or %	Number or count/available
Intended population	70.4	57
Age	65 (54–80)	57
Prebiopsy serum PSA (ng/ml)	9.59 (1.08–19.45)	57
1–10 ng/ml	59.6	34/57
10–20 ng/ml	40.4	23/57
Suspicious DRE	30	11/37
No prior biopsy	91.2	52/57
Number of cores	10	57
Positive biopsy result	24.6	14/57
Gleason score		
GS = 6 (3 + 3)	10.5	6/57
GS ≥ 7	14.03	8/57
GS = 7 (3 + 4)	3.5	2/57
GS = 7 (4 + 3)	5.3	3/57
GS = 9 (5 + 4)	1.8	1/57
GS = 10 (5 + 5)	3.5	2/57

**Table 2:** LBXexo in prediction of all biopsies, evaluated by the Gleason score.

	Biopsy result					
	Negative (*n* = 43)	GS = 6 (*n* = 6)	GS = 3 + 4 (*n* = 2)	GS = 4 + 3 (*n* = 3)	GS = 5 + 4 (*n* = 1)	GS = 5 + 5 (*n* = 2)
LBXexo score						
Positive	17	4	2	3	1	2
Negative	26	2	0	0	0	0
Positive percentage	39.5	66.7	100	100	100	100

**Table 3:** Performance of PCA3 and LBXexo in predicting biopsy outcomes.

Any-grade cancer	Biopsy positive	Biopsy negative	Total	Performance	
PCA3					
≥Cutoff point	10	28	38	PPV %	26.32
<Cutoff point	4	15	19	NPV %	78.95
Total	14	43	57		
LBXexo score					
≥Cutoff point	12	17	29	PPV %	41.38
<Cutoff point	2	26	28	NPV %	92.86
Total	14	43	57		
	Sensitivity %	Specificity %	% of predicted negative		
PCA3	71.4	34.9	33.33		
LBXexo	85.7	60.5	50.88		

High-grade cancer (GS ≥ 7)	Biopsy positive	Biopsy negative + low-grade cancer	Total	Performance	

PCA3					
≥Cutoff point	7	31	38	PPV %	18.42
<Cutoff point	1	18	19	NPV %	94.74
Total	8	49	57		
LBXexo score					
≥Cutoff point	8	21	29	PPV %	27.59
<Cutoff point	0	28	28	NPV %	100
Total	8	49	57		
	Sensitivity %	Specificity %	% of predicted negative		
PCA3	87.5	36.7	33.33		
LBXexo	100	57.1	49.12		

Abbreviations: PPV, positive predictive value; NPV, negative predictive value.

**Table 4:** Diagnostic performances of PCA3 and LBXexo in predicting biopsy outcomes.

Parameter	AUC	95% CI	*p*
Any-grade cancer			
PSA	0.595	0.436–0.754	0.289
PCA3	0.561	0.38–0.746	0.479
LBXexo score	0.723	0.58–0.849	0.017^*∗*^

High-grade cancer			
PSA	0.529	0.334–0.723	0.797
PCA3	0.703	0.511–0.899	0.065
LBXexo score	0.736	0.592–0.868	0.038^*∗*^

Abbreviations: CI, confidence interval; AUC, area under the curve. ^*∗*^*p* < 0.05.

**Table 5:** LBXexo in predictions of all biopsies, evaluated by the Gleason score in the validation cohort.

	Biopsy Result					
	Negative (*n* = 17)	GS = 6 (*n* = 5)	GS = 3 + 4 (*n* = 3)	GS = 4 + 3 (*n* = 1)	GS = 4 + 4 (*n* = 1)	GS = 5 + 4 (*n* = 1)
LBXexo score						
Positive	11	5	3	1	1	1
Negative	6	0	0	0	0	0
Positive percentage	64.7	100	100	100	100	100

## Data Availability

All data generated or analyzed during this study are included within the article and its additional files.

## Abbreviations

PCa: Prostate cancer
DRE: Digital rectal exam
PCA3: Prostate cancer antigen 3
PRAC: Prostate cancer susceptibility candidate
GS: Gleason score
ROC: Receiver-operating characteristic
AU: Area under the curve
PPV: Positive predictive value
NPV: Negative predictive value
CI: Confidence interval.

## Supplementary Materials

Supplementary Materials Supplementary Table 1: subjects' clinical characteristics in the validation cohort. .

## References

[1] Bray F., Ferlay J., Soerjomataram I., Siegel R. L., Torre L. A., Jemal A. (2018). Global cancer statistics 2018: GLOBOCAN estimates of incidence and mortality worldwide for 36 cancers in 185 countries. *CA: A Cancer Journal for Clinicians*.

[2] Chen W., Zheng R., Baade P. D. (2016). Cancer statistics in China, 2015. *CA: A Cancer Journal for Clinicians*.

[3] Chen R., Ren S., Yiu M. K. (2014). Prostate cancer in Asia: a collaborative report. *Asian Jounal of Urology*.

[4] Djavan B., Zlotta A., Remzi M. (2000). Optimal predictors of prostate cancer on repeat prostate biopsy. *Journal of Urology*.

[5] Loeb S., Vellekoop A., Ahmed H. U. (2013). Systematic review of complications of prostate biopsy. *European Urology*.

[6] Catalona W. J., Partin A. W., Slawin K. M. (1998). Use of the percentage of free prostate-specific antigen to enhance differentiation of prostate cancer from benign prostatic disease. *JAMA: The Journal of the American Medical Association*.

[7] Pearson J. D., Luderer A. A., Jeffrey Metter E. (1996). Longitudinal analysis of serial measurements of free and total PSA among men with and without prostatic cancer. *Urology*.

[8] Carter H. B., Pearson J. D., Metter E. J. (1992). Longitudinal evaluation of prostate-specific antigen levels in men with and without prostate disease. *JAMA: The Journal of the American Medical Association*.

[9] Sokoll L. J., Sanda M. G., Feng Z. (2010). A prospective, multicenter, National Cancer Institute early detection research network study of [−2]proPSA: improving prostate cancer detection and correlating with cancer aggressiveness. *Cancer Epidemiology Biomarkers & Prevention*.

[10] Wang W., Wang M., Wang L., Adams T. S., Tian Y., Xu J. (2014). Diagnostic ability of % p2PSA and prostate health index for aggressive prostate cancer: a meta-analysis. *Scientific reports*.

[11] Hessels D., Klein Gunnewiek J. M. T., van Oort I. (2003). DD3PCA3-based molecular urine analysis for the diagnosis of prostate cancer. *European Urology*.

[12] Day J. R., Jost M., Reynolds M. A., Groskopf J., Rittenhouse H. (2011). PCA3: from basic molecular science to the clinical lab. *Cancer Letters*.

[13] Nakanishi H., Groskopf J., Fritsche H. A. (2008). PCA3 molecular urine assay correlates with prostate cancer tumor volume: implication in selecting candidates for active surveillance. *Journal of Urology*.

[14] Wei J. T., Feng Z., Partin A. W. (2014). Can urinary PCA3 supplement PSA in the early detection of prostate cancer?. *Journal of Clinical Oncology*.

[15] Durand X., Xylinas E., Radulescu C. (2012). The value of urinary prostate cancer gene 3 (PCA3) scores in predicting pathological features at radical prostatectomy. *BJU International*.

[16] Auprich M., Chun F. K.-H., Ward J. F. (2011). Critical assessment of preoperative urinary prostate cancer antigen 3 on the accuracy of prostate cancer staging. *European Urology*.

[17] Tomlins S. A., Rhodes D. R., Perner S. (2005). Recurrent fusion of TMPRSS2 and ETS transcription factor genes in prostate cancer. *Science*.

[18] Magi-Galluzzi C., Tsusuki T., Elson P. (2011). TMPRSS2-ERG gene fusion prevalence and class are significantly different in prostate cancer of Caucasian, African-American and Japanese patients. *The Prostate*.

[19] Leyten G. H. J. M., Hessels D., Jannink S. A. (2014). Prospective multicentre evaluation of PCA3 and TMPRSS2-ERG gene fusions as diagnostic and prognostic urinary biomarkers for prostate cancer. *European Urology*.

[20] Hessels D., Smit F. P., Verhaegh G. W., Witjes J. A., Cornel E. B., Schalken J. A. (2007). Detection of TMPRSS2-ERG fusion transcripts and prostate cancer antigen 3 in urinary sediments may improve diagnosis of prostate cancer. *Clinical Cancer Research*.

[21] De Toro J., Herschlik L., Waldner C., Mongini C. (2015). Emerging roles of exosomes in normal and pathological conditions: new insights for diagnosis and therapeutic applications. *Frontiers in Immunology*.

[22] Momen-Heravi F., Getting S. J., Moschos S. A. (2018). Extracellular vesicles and their nucleic acids for biomarker discovery. *Pharmacology & Therapeutics*.

[23] Verma M., Lam T. K., Hebert E., Divi R. L. (2015). Extracellular vesicles: potential applications in cancer diagnosis, prognosis, and epidemiology. *BMC Clinical Pathology*.

[24] Donovan M. J., Noerholm M., Bentink S. (2015). A molecular signature of PCA3 and ERG exosomal RNA from non-DRE urine is predictive of initial prostate biopsy result. *Prostate Cancer and Prostatic Diseases*.

[25] Nilsson J., Skog J., Nordstrand A. (2009). Prostate cancer-derived urine exosomes: a novel approach to biomarkers for prostate cancer. *British Journal of Cancer*.

[26] McKiernan J., Donovan M. J., O’Neill V. (2016). A novel urine exosome gene expression assay to predict high-grade prostate cancer at initial biopsy. *JAMA Oncology*.

[27] McKiernan J., Donovan M. J., Margolis E. (2018). A prospective adaptive utility trial to validate performance of a novel urine exosome gene expression assay to predict high-grade prostate cancer in patients with prostate-specific antigen 2–10 ng/ml at initial biopsy. *European Urology*.

[28] Ren S., Peng Z., Mao J.-H. (2012). RNA-seq analysis of prostate cancer in the Chinese population identifies recurrent gene fusions, cancer-associated long noncoding RNAs and aberrant alternative splicings. *Cell Research*.

[29] Lenka G., Weng W.-H., Chuang C.-K., Ng K.-F., Pang S.-T. (2013). Aberrant expression of the PRAC gene in prostate cancer. *International Journal of Oncology*.

[30] Théry C., Amigorena S., Raposo G., Clayton A. (2006). Isolation and characterization of exosomes from cell culture supernatants and biological fluids. *Current Protocols in Cell Biology*.

[31] Thompson I. M., Pauler D. K., Goodman P. J. (2004). Prevalence of prostate cancer among men with a prostate-specific antigen level ≤4.0 ng per milliliter. *New England Journal of Medicine*.

[32] Draisma G., Etzioni R., Tsodikov A. (2009). Lead time and overdiagnosis in prostate-specific antigen screening: importance of methods and context. *JNCI Journal of the National Cancer Institute*.

[33] Gulati R., Inoue L. Y. T., Gore J. L., Katcher J., Etzioni R. (2014). Individualized estimates of overdiagnosis in screen-detected prostate cancer. *JNCI Journal of the National Cancer Institute*.

[34] Macefield R. C., Metcalfe C., Lane J. A. (2010). Impact of prostate cancer testing: an evaluation of the emotional consequences of a negative biopsy result. *British Journal of Cancer*.

[35] Huang M., Lin Y., Xu A. (2014). Percent free prostate-specific antigen does not improve the effectiveness of prostate cancer detection in Chinese men with a prostate-specific antigen of 2.5–20.0 ng/ml: a multicenter study. *Medical Oncology*.

[36] Morote J., Trilla E., Esquena S. (2002). The percentage of free prostatic-specific antigen is also useful in men with normal digital rectal examination and serum prostatic-specific antigen between 10.1 and 20 ng/ml. *European Urology*.

[37] Li M., Na Y. Q. (2008). The detection rate of prostate cancer in different prostate-specific antigen (PSA) levels in Chinese men. *Zhonghua Yi Xue Za Zhi*.

[38] Wu Y. S., Na R., Xu J. F., Bai P. D., Jiang H. W., Ding Q. (2014). The influence of prostate volume on cancer detection in the Chinese population. *Asian Journal of Andrology*.

[39] Cui Y., Cao W., Li Q. (2016). Evaluation of prostate cancer antigen 3 for detecting prostate cancer: a systematic review and meta-analysis. *Scientific Reports*.

[40] Stamatiou K., Alevizos A., Karanasiou V. (2007). Impact of additional sampling in the TRUS-guided biopsy for the diagnosis of prostate cancer. *Urologia Internationalis*.

[41] Isbarn H., Briganti A., De Visschere P. J. (2015). Systematic ultrasound-guided saturation and template biopsy of the prostate: indications and advantages of extended sampling. *Archivos Espanoles de Urologia*.

[42] Raposo G., Stoorvogel W. (2013). Extracellular vesicles: exosomes, microvesicles, and friends. *The Journal of Cell Biology*.

[43] Harding C. V., Heuser J. E., Stahl P. D. (2013). Exosomes: looking back three decades and into the future. *The Journal of Cell Biology*.

[44] Minciacchi V. R., Freeman M. R., Di Vizio D. (2015). Extracellular vesicles in cancer: exosomes, microvesicles and the emerging role of large oncosomes. *Seminars in Cell & Developmental Biology*.

[45] Vader P., Breakefield X. O., Wood M. J. A. (2014). Extracellular vesicles: emerging targets for cancer therapy. *Trends in Molecular Medicine*.

[46] Minciacchi V. R., Zijlstra A., Rubin M. A., Di Vizio D. (2017). Extracellular vesicles for liquid biopsy in prostate cancer: where are we and where are we headed?. *Prostate Cancer and Prostatic Diseases*.

[47] Del Re M., Biasco E., Crucitta S. (2017). The detection of androgen receptor splice variant 7 in plasma-derived exosomal RNA strongly predicts resistance to hormonal therapy in metastatic prostate cancer patients. *European Urology*.

[48] Kato T., Mizutani K., Kameyama K. (2015). Serum exosomal P-glycoprotein is a potential marker to diagnose docetaxel resistance and select a taxoid for patients with prostate cancer. *Urologic Oncology: Seminars and Original Investigations*.

[49] Kim M. S., Haney M. J., Zhao Y. (2016). Development of exosome-encapsulated paclitaxel to overcome MDR in cancer cells. *Nanomedicine: Nanotechnology, Biology and Medicine*.

