# Supplementary material for: Detection of Prostate Cancer Antigen 3 and Prostate Cancer Susceptibility Candidate in Non-DRE Urine Improves Diagnosis of Prostate Cancer in Chinese Population
Source: Prostate Cancer. 2020 Jan 31;2020:3964615. doi: 10.1155/2020/3964615 (PMC7013283; doi:10.1155/2020/3964615)
Supplement: Supplementary Materials — Supplementary Table 1: subjects' clinical characteristics in the validation cohort. . [file 3964615.f1.pdf]

Detection of Prostate Cancer Antigen 3 and Prostate Cancer Susceptibility Candidate in Non-DRE urine Improves Diagnosis of Prostate Cancer in Chinese

Lie-Fu Ye[1, 2], Sha He[2], Xiaopei Wu[3], Shengying Jiang[3], Ruo-Chen Zhang[1], Ze-Song Yang[1], Fa-Wen Chen[4], Dan-Ling Pan[5], Dong Li[6], Gang Li[3]

Running title: exoPCA3/PRAC improves diagnosis of prostate cancer in Chinese

[1]Department of Urology, Fujian Provincial Hospital, Fuzhou, Fujian, [2]Fujian Medical University Provincial Clinical College, Fuzhou, Fujian, [3]Fuzhou L-BioMedx Technology Co., Ltd., Fuzhou, Fujian, [4]Clinical Laboratory, Fujian Provincial Hospital, Fuzhou, Fujian, [5]Department of Pathology, Fujian Provincial Hospital, Fuzhou, Fujian, [6]School of Medicine, Nankai University, Tianjin, China

L. Ye and S. He contributed equally to this paper.

Address Correspondence to: Gang Li, Fuzhou L-BioMedx Technology Co., Ltd., B-822, Building 10, Haixiyuan, Gaoxin District, Fuzhou, Fujian, China 350108

Phone: 086-591-62085629

Fax: 086-591-62085629

E-mail: gli9809@gmail.com

Emails for other co-authors:

Lie-Fu Ye yeliefu@126.com

Sha He 1121262902@qq.com

Xiaopei Wu lbs_wuxp@163.com

Shengying Jiang 1805961889@qq.com

Ruo-Chen Zhang 441374690@qq.com

Ze-Song Yang 33320298@qq.com

Fa-Wen Chen chenfawen@163.com

Dan-Ling Pan pandanling0909@sina.com

Dong Li lidong@nankai.edu.cn

Suppl Table 1. Subjects Clinical Characteristics in Validation Cohort

Characteristic	Median (range) or %	Number (count/available)
Intended use population	100%	28
Age	65 (51-810)	28
Pre-biopsy serum PSA (ng/ml)	10.32 (4.8-17.91)	28
1-10ng/ml	46.4%	13/28
10-20ng/ml	53.6%	15/28
Suspicious DRE	10%	1/10
No prior biopsy	89.3%	25/28
Median number of cores	10 (10-12)	28
Biopsy result positive	39.3%	11/28
Gleason score		
GS=6 (3+3)	17.9%	5/28
GS≥7	21.4%	6/28
GS=7 (3+4)	10.7%	3/28
GS=7 (4+3)	3.6%	1/28
GS=8 (4+4)	3.6%	1/28
GS=9 (5+4)	3.6%	1/28
